# Responsiveness and minimal important differences for patient reported outcomes

**DOI:** 10.1186/1477-7525-4-70

**Published:** 2006-09-27

**Authors:** Dennis A Revicki, David Cella, Ron D Hays, Jeff A Sloan, William R Lenderking, Neil K Aaronson

**Affiliations:** 1 Center for Health Outcomes Research, United Biosource Corporation, 7101 Wisconsin Ave., Suite 600, Bethesda, MD 20814, USA; 2 Evanston Northwestern Healthcare, Center on Outcomes Research and Education, Evanston, IL, USA; 3 UCLA Division of General Internal Medicine and Health Services Research, 911 Broxton Plaza, Room 110, Los Angeles, CA, 90024, USA; 4 Department of Health Sciences Research, Mayo Clinic, 200 First Street SW, Rochester, MN, 55905, USA; 5 Worldwide Outcomes Research, Pfizer Inc., Eastern Point Road, Groton, CT 06340, USA; 6 Division of Psychosocial Research and Epidemiology, The Netherlands Cancer Institute, Plesmanlaan 121, 1066CX Amsterdam, The Netherlands

## Abstract

Patient reported outcomes provide the patient's perspective on the effectiveness of treatment. The draft Food and Drug Administration guidance on patient reported outcomes for labeling and promotional claims raises a number of method and measurement issues that require further clarification, including methods of determining responsiveness and minimal important differences. For clinical trials, instruments need to be based on a clear conceptual framework, have evidence supporting content validity and acceptable psychometric qualities. The measures must also have evidence documenting responsiveness and interpretation guidelines (i.e., minimal important difference) to be most useful as effectiveness endpoints in clinical trials. The recommended approach is to estimate the minimal important difference based on several anchor-based methods, with relevant clinical or patient-based indicators, and to examine various distribution-based estimates (i.e., effect size, standardized response mean, standard error of measurement) as supportive information, and then to triangulate on a single value or small range of values for the MID. Confidence in a specific MID value evolves over time and is confirmed by additional research evidence, including clinical trial experience. The MID may vary by population and context, and no one MID will be valid for all study applications involving a PRO instrument. Responsiveness and MID must be demonstrated and documented for the particular study population, and these measurement characteristics are needed for PRO labeling and promotional claims.

## Introduction

Patient reported outcomes (PROs) provide the patient's perspective on the effectiveness of treatment, and for many diseases the patient is really the only source of health outcome endpoint data [1-3]. The draft FDA guidance on PROs for labeling and promotional claims raises a number of method and measurement issues that require further clarification [4]. For clinical trials evaluating new pharmaceuticals, PRO instruments need to be based on a clear conceptual framework, have evidence supporting content validity (i.e., the instrument content reflects the key characteristics of the construct from the patient's perspective), and must have demonstrated acceptable psychometric qualities (e.g., reliability, validity) [1,2]. The PRO measures must also have evidence documenting responsiveness or sensitivity to changes in clinical status to be most useful as effectiveness endpoints in clinical trials. Without evidence that the PRO can detect meaningful changes in health status, using the PRO in a clinical trial may be risky, because clinically meaningful effects may go undetected. Responsiveness is an aspect of construct validity and is determined by evaluating the relationship between changes in clinical and other endpoints and changes in the PRO scores over time, or based on the application of a treatment of known and demonstrated efficacy, in either observational studies or in clinical trials [2,5,6].

Demonstrating responsiveness is necessary, but additional information is needed to determine the minimally important difference (MID) for a PRO measure. Responsiveness represents the instrument's ability to detect changes in health status while MID is used to interpret whether the observed change is important from the patient's or clinician's perspective. Increasingly, in health outcomes research the MID is based primarily on the patient's perspective with the clinician's viewpoint serving to confirm the findings on MID. Responsiveness and MID vary by population and contextual characteristics, and there is no single MID value for a PRO instrument across all applications and patient samples. Once that range in MIDs is determined, one can decided which particular value to use as a basis for sample size calculation.

The MID has been defined as the smallest change in a PRO measure that is perceived by patients as beneficial or that would result in a change in treatment [5,7]. There are a number of anchor-based and distribution-based methods that have been used to determine the MID for PRO measures [7-9]. The anchor-based methods require an external patient-based or clinical criteria to inform as to changes in PRO scores that are meaningful. The distribution-based methods reflect one or several statistical indices of change. However, the current situation for determining the MID is fluid, but there is an evolving consensus as to the recommended, best practice methods for determining the MID [7].

The recommended approach is to estimate the MID based on several anchor-based methods, with relevant clinical or patient-based indicators, and to examine various distribution-based estimates (i.e., effect size, standardized response mean, standard error of measurement) as supportive information, and then to triangulate on a single value or small range of values for the MID. Confidence in a specific MID value evolves over time and is confirmed by additional research evidence, including clinical trial experience. It must be recognized and accepted that aspects of PRO assessment include some measurement error and that no PRO measure is error free and should not be expected to be so in order to be used in clinical trials. There does however need to be evidence that the psychometric characteristics of the PRO instrument are such that there is confidence that changes in scores over time with the application of treatments with some efficacy can be detected [10] and that the measurement error (or noise) is not so large that it is problematic to observe meaningful changes in patients' health status.

## Assessing the responsiveness of PRO instruments

Longitudinal studies are needed to determine whether a PRO instrument is responsive to changes or differences in health status. These studies may be randomized clinical trials comparing treatments of known efficacy or observational studies where patients are treated with usual medical care and followed over relevant periods of time. To assess responsiveness, some criterion is needed to identify whether patients have changed (either improved or worsened) over time. These criteria, or anchors, may be clinical endpoints (i.e., laboratory measures, physiological measures, clinician ratings), patient-rated global improvement or other PROs with established responsiveness, or some combination of clinical and patient-based outcomes. The anchor-based approaches use an external indicator, either clinical or patient-based, to assign subjects into several groupings reflecting no change, small positive changes, large positive changes, small negative changes, or large negative changes in clinical or health status. It is highly recommended to use multiple independent anchors and to examine and confirm responsiveness across multiple samples.

Selecting anchors should be based on criteria of relevance for the disease indication, clinical acceptance and validity, and evidence that the anchors have some relationship with the PRO measure. It is recommended that researchers determine the strength of the association of the anchor measure with the PRO. An anchor that has a very low or no correlation with the PRO instrument may provide misleading information in determining whether significant change has occurred. There also needs to be an understanding of the trajectory of health outcomes in the target disease to evaluate responsiveness. For example, do most patients improve over time with treatment, as with seasonal allergic rhinitis or, as in many chronic diseases (e.g., COPD, arthritis, etc.) is the expected trajectory one of maintenance of health status versus varying levels of deterioration in health status over time, even with treatment?

Once groups of patients are identified as improving, worsening or remaining stable based on several relevant external anchors, several types of data analysis and indicators can be used to examine responsiveness. First, analysis of variance or covariance procedures can be performed comparing differences in mean baseline to endpoint changes in the PRO scores across the meaningful change groups (i.e., stable versus small improvement, stable versus moderate improvement, etc.). Second, responsiveness to change is frequently evaluated using different indicators [6,10], such as the effect size (ES) [11], standardized response mean (SRM) [12], and the responsiveness statistic (RS) [5]. For these three indices, the numerator is the mean baseline to endpoint change and the denominators are the standard deviation (SD) at baseline (ES), the SD of change for the group (SRM), or the SD of change in patients that remain stable over time (RS). For the ES, Cohen [13] provided guidance on interpretation of the magnitude, where a 0.20 ES is considered a small change, 0.50 is viewed a moderate change, and 0.80 is viewed as a large change.

Some researchers have suggested that the 1/2 standard deviation rule [14] or that the standard error of measurement (SEM) [15,16] may represent the MID for PRO instruments. While this magnitude of change is certainly clinically significant and important, since in the case of the 1/2 SD this represents a moderate effect size [13], it may not be the smallest nonignorable difference. These differences in PRO scores are just too large to be considered minimally important. While these different distribution-based indicators demonstrate that change has occurred and provide some insight as to whether the change (responsiveness) is small or large, the indices do not necessarily inform as to whether the observed change in MID. To determine MID, it is necessary to get information as to whether the observed change in important from the patient's or clinician's perspective [17]. Based on these methods, MIDs can be in the range of 0.20 to 0.30 ES (or SD units).

## Determining the MID for PRO instruments

For interpreting differences or changes in PRO instruments, information needs to be provided as to whether the changes seen in the scores are important from either the patient's or clinician's perspective. The clinical meaningfulness of the observed change is based on that change being perceived as minimally important and that would be perceived as beneficial from the patient's viewpoint. It is recommended that the patient's perspective be given the most weight, since these are PROs, although the clinician's perspective is considered important as well. The MID is determined based on multiple anchors, that is the same external criteria used to evaluate responsiveness of the PRO measure. However, there are differences in how these data are used and compared to determine MID. Since the focus is on determining the MID, it is necessary to identify the smallest difference or change that is important to the patient.

In many cases, global assessments of change in health or clinical status are used to categorize patients into groups that reflect, based on their own reports, different amounts of change in the construct of interest. For example, based on the Overall Treatment Effect (OTE) scale [18], patients can be assigned into groups representing no change (i.e., remaining stable), small improvements, moderate improvements or large improvements, and small amount of worsening, moderate worsening, or large amounts of worsening. The MID is viewed as the observed change seen in the small improvement group, if this change is larger than that seen in the stable group. If is some variation observed among the stable group, the MID may be based on the difference in mean baseline to endpoint change scores between the stable group and the small improvement (or worsening) group. Note that there is evidence that there is asymmetry in worsening and improvement in PROs depending on the specific disease [19,20]. Equally, clinician global assessments of change in clinical status or evaluations of clinical severity, clinical response criteria (i.e., ACR response criteria) or other indicators can be used to determine MID. For these clinical anchors, it will be necessary to identify, based on previous research or clinical consensus, what a small and clinically meaningful effect may be, based on these measures. For example, in rheumatoid arthritis, the differences between groups of stable patients and those experiencing a 20% ACR response can be used to determine the MID of a PRO score. If multiple anchors are used, there will be several different estimates of MID derived corresponding to these different anchors, and the result will be a range of MID estimates for the targeted PRO instrument.

Finally, the application of multiple methods to determine the MID for a PRO instrument in a specific patient population will result in a range of values for the MID. This is the essence of triangulation, that is, examining multiple values from different approaches and hopefully converging on a small range of values (or one single value). It is recommended that the different MID estimates be first graphed to visually depict the range of estimates. To identify a single MID value (or narrow range of MID values), it is recommended that the anchor-based estimates be assigned the most weight and experience from clinical trials be used to further support and perhaps further narrow the range of values. Care must be taken in selecting the most appropriate anchors, as measurement error can be magnified if the anchors are not measured reliably. Interpretation of the MID from different anchors should also take into account the proximity of the anchor to the target PRO measure, that is, assign more importance to MIDs generated from more closely linked concepts. A systematic consensus process involving several clinicians and health outcome researchers is recommended and can be completed, based on Delphi methods, to arrive at a single MID value, or at least a narrower range of values. There is no consensus as to how much data are needed as supportive evidence for the MID of a PRO instrument. Clearly, the more data and evidence the better, but a single, generalizable study with multiple patient-based and clinical anchors may be sufficient.

As with other aspects of construct validity, responsiveness and the MID value are confirmed based on accumulating evidence from multiple studies and, with additional data, we can be more confident in the MID value. A single MID cannot be assumed to be appropriate for all applications and across all patient populations; it is unlikely that this will be the case. For example, the MID derived for an asthma-specific quality of life measure in mild to moderate asthma patients may not be generalizable to clinical trials comparing an add-on treatment for patients with moderate to severe asthma [21]. Finally, it may not always be feasible or practical to identify anchors for all PRO assessments, in such cases, distribution-based approaches to calculating the MID can still provide some guidance for decision-making. Until further evidence is obtained regarding the relative utility and veracity of competing approaches for estimating an MID, it is likely that the optimal approach will be study-specific.

## Conclusion

For PRO endpoint data to be accepted as evidence of treatment effectiveness, there must be evidence documenting the instrument's conceptual framework, content validity, and psychometric qualities, including reliability, validity and responsiveness. For responsiveness, it is necessary to demonstrate that the PRO scores are sensitive to actual changes in clinical or health status. While demonstrating responsiveness is a key component to establishing an instrument's construct validity, it is also important to determine the MID to assist in interpreting statistical significant PRO results in clinical trials. The MID may vary by population and context, and no one MID will be valid for all study applications involving a PRO instrument. Responsiveness and MID must be demonstrated and documented for the particular study population, and these measurement characteristics are needed for PRO labeling and promotional claims.

## Competing interests

The author(s) declare that they have no competing interests.

## Authors' contributions

All of the authors contributed to the conceptualization, contributed content and participated in the development of the final manuscript. All authors read and approved the final manuscript.
